# A Systematic Review of Momentary Assessment Designs for Mood and Anxiety Symptoms

**DOI:** 10.3389/fpsyg.2021.642044

**Published:** 2021-05-17

**Authors:** Mila Hall, Paloma V. Scherner, Yannic Kreidel, Julian A. Rubel

**Affiliations:** Psychotherapy Research Lab, Department of Psychology, Justus-Liebig University Giessen, Giessen, Germany

**Keywords:** ecological momentary assessment, intensive longitudinal methods, depression, anxiety, methodological review

## Abstract

**Background:** Altering components of ecological momentary assessment (EMA) measures to better suit the purposes of individual studies is a common and oftentimes necessary step. Though the inherent flexibility in EMA has its benefits, no resource exists to provide an overview of the variability in how convergent constructs and symptoms have been assessed in the past. The present study fills that gap by examining EMA measurement design for mood and anxiety symptomatology.

**Methods:** Various search engines were used to identify 234 relevant studies. Items administered, data collection schedules (i.e., beeps per day), response scales (i.e., Likert), data collection platforms (i.e., apps), and psychometric properties (i.e., reliability) were extracted.

**Results:** Study designs varied greatly in all aspects across the identified papers. Over 4,600 extracted items were qualitatively analyzed, resulting in the identification of 12 themes. The most EMA items focused on affect, with categories such as “happiness” and “tension” appearing most frequently. We provide all of our data extraction in the format of an open-source database.

**Limitations:** Despite our best attempts to include as much of the relevant literature as possible, this review and the accompanying database are not exhaustive, but can easily be built upon to include other, newer studies.

**Conclusions:** The fact that the affect theme featured both positive and negative emotional constructs highlights the dichotomous focus on valence and affect within the literature surrounding anxious and depressive symptomatology. We hope that our database will act as a helpful design decision-making resource for researchers studying this kind of symptomatology in the future.

**Systematic Review Registration:** PROSPERO (CRD42019139409).

## Introduction

The concept of daily-life studies is not new, indicated by the fact that methods can be traced back to the period following World War II, when governments were interested in learning about the general population's leisure activities (Wheeler and Reis, 1991). Generally speaking, these methods aim to study daily life, typically by measuring constructs multiple times per day for an extended period of time. These methods have grown in popularity across various fields of psychology (Armey et al., 2015) in part due to the widespread use and increasing time spent on portable electronic devices (Trull and Ebner-Priemer, 2014; Hitlin, 2018). These daily life-focused methods have elicited burgeoning interest within areas of clinical psychology, in particular for the study of mood and anxiety disorders (e.g., Fisher et al., 2017).

Though various names have been given to this kind of research design (i.e., experience sampling, daily diaries, intensive longitudinal data collection), we will refer to all of these as ecological momentary assessment (EMA). Overall, EMA describes a type of data collection that allows researchers to gain detailed insight into the daily lives of participants. Often, EMA items inquire about the participants' mental state in the moment (Haedt-Matt and Keel, 2011) in an effort to avoid memory recall bias (Fahrenberg et al., 2007), which many retrospective assessments struggle with (Gorin and Stone, 2001). Given how flexible this method is in allowing researchers to tailor measurement schedules, item phrasing, and many more aspects to their specific research question, it is unsurprising that the method's implementation has been quite varied.

### Methodological Variation in EMA Research

There are numerous ways in which EMA data collection can differ from study to study, creating a large amount of variation in the design and implementation of such studies. First, there are three broad categories of data collection schedules to consider: (a) signal-contingent: participants respond to questionnaires when they are pinged (i.e., four times per day at random times), (b) interval-contingent: participants respond to pings that are spaced out using predetermined time intervals (i.e., receiving a ping every 3 h), and (c) event-contingent: participants fill out questionnaires every time a specific event occurs (e.g., when they experience a panic attack; Wheeler and Reis, 1991). For the purposes of this study, we include signals related to specific contexts as part of event-contingent signaling. For example, if a participant enters into a particular social or physical context (e.g., starting a conversation with other people or walking into a bar, respectively), this is considered to be an “event.” Each of these schedules address different kinds of research questions. Interval- and signal-contingent recording are beneficial when it comes to examining constructs that are dynamic over time, while event-contingent recording can be used to study specific situations (e.g., during panic attacks; Sakamoto et al., 2008; Walz et al., 2014). Despite the fact that each method has its benefits and may be particularly well-suited to address specific research questions, the differences in the timing of the pings can complicate the generalizability of findings across EMA studies. More specifically, researchers must be cautious in comparing results across different types of schedules (e.g., event- vs. signal-contingent) since they may fundamentally be studying divergent aspects of a psychological condition: one may be focused on the frequency and intensity of panic attacks throughout the week, while another may be more interested in more granular fluctuations in anxiety on one specific day. Findings from these two hypothetical studies may support one another from a theoretical standpoint, but generalizing results between them would be unwise since they measure different aspects of anxiety.

When implementing EMA methods, using questionnaires designed and validated for cross-sectional data collection is also a common yet questionable research practice (Flake and Fried, 2019). These types of questionnaires may not be appropriate or transferable to an intensive longitudinal design (Moskowitz et al., 2009). For example, if a cross-sectionally validated questionnaire asks about weekly symptoms, administering this questionnaire multiple times per day would render its validity questionable (Hufford, 2007). Stone and colleagues additionally report that different temporal contextualizations in the instructions (e.g., *today* vs. *in the last day*) led to a difference in reported affective states (Stone et al., 2020). These differences may be subtle, but indicate that even small sources of measurement heterogeneity can have wide-reaching consequences. Providing clear and intentionally-selected timeframes for items, remaining consistent with them throughout the questionnaire, is therefore recommended to avoid confusion and misinterpretation by the participant. Participants additionally seem to have difficulties delineating some experiences to particular hours within in a day, therefore making it more straightforward to ask about the day as a whole instead of a specific timeframe within a day (e.g., using *today* instead of *within the last 3 h*). It may be easier to identify whether more concrete occurrences happened within a specific timeframe (e.g., *Within the last 3 h, I interacted with a family member*) compared to more subtle experiences which may be better measured over the course of a whole day (e.g., *How melancholic did you feel today?*). Interestingly, a study by Schuler et al. (2019) found no significant differences between the retrospective reports of symptoms when compared to EMA-based reports of traumatic experiences. These findings may reflect the unique circumstances involved in recall of such experiences, which are inherently more emotionally extreme and may therefore be easier to recall, even retrospectively. Making these kinds of methodological design decisions in EMA studies must be based on whether it will facilitate the validity and reliability of the questions, in addition to any theoretical assumptions. Based on qualitative interviews with EMA researchers, it appears that reliability, validity, and theory are in fact significant priorities in the design decision-making process (Janssens et al., 2018). However, the extent to which these are prioritized and enacted from a methodological perspective (e.g., by calculating within-person validity and reliability indices as part of the research process) is unclear.

Transferring a questionnaire, which was originally designed for paper-pencil use, to a computerized version of the same assessment is also potentially problematic. Shifting an originally paper-pencil questionnaire to a digital format could mean that items may need reformatting to be suitable for computer use, the response scale may need to be adjusted, or even just interacting with a computer as opposed to manually writing may have an influence on ratings. While these are valid points of criticism, a meta-analysis showed that written and computerized assessments have equivalent outcomes for patient-reported data (Gwaltney et al., 2008). Perhaps the limited transferability and comparability of paper-pencil questionnaires to digital formats are less critical (Gwaltney et al., 2008). Since comparability across these formats can be assumed, researchers should take advantage of the benefits that accompany the use of computerized assessments (e.g., higher compliance, less time spent entering, and checking data manually; Hufford, 2007).

Nonetheless, the variability in how measures are administered due to a need to shorten and alter items to make them more suitable for mobile administration should be noted. Though the aforementioned meta-analysis found no differences in patient reports across paper-pencil and computerized questionnaires, they cannot be assumed to be equivalent. The recommendation should not be to simply transfer and freely alter a previously validated paper-pencil questionnaire to better serve the purposes of a single study without further inspection of the effects those changes may have on the reported outcomes (Flake and Fried, 2019; Stone et al., 2020). A failure to acknowledge these shortcomings puts the validity, reliability and replicability of EMA studies at risk.

### EMA Measurement of Mood and Anxiety

Given the exciting and novel opportunities for research using EMA, alongside the flexibility with which it can be implemented, there has been steady growth within mood and anxiety research. The methodological differences across these studies, while partially a reflection of the myriad of research questions in this area, has also meant that comparing results across studies is harder. For example, a 2012 review of EMA studies of Major Depressive Disorder (MDD) showed that the total duration of EMA data collection ranged from 3 to 42 days, with daily assessments occurring anywhere between 2 to 10 times (aan het Rot et al., 2012). Though this may be, in part, a reflection of how different aspects of MDD are expected to operate with differing speeds and over different periods of time, the variability in this measurement complicates comparison across these studies.

Another systematic review of EMA and its use for studying anxiety disorders, such as Generalized Anxiety Disorder (GAD), identified an even larger range of measurement variability: people with GAD were measured anywhere between 2 to 140 days, with pings per day ranging from 2 to 16 times per day (Walz et al., 2014). Once again, GAD is a complex syndrome of which different aspects may evolve at different paces. However, this once again means that these studies will be more difficult to compare, summarize, and merge into a comprehensive picture of GAD or its components.

These systematic reviews provide a glimpse into the extent to which EMA studies have heterogeneous designs. In addition to the fact that comparing results across studies with different focuses and designs, another source of variation across these studies is the specific items used to assess mood and anxiety-related variables. Though open science practices, such as providing supplemental materials with full lists of the items administered, are growing in popularity, these resources are provided inconsistently and, for older EMA studies, may not be available at all anymore (Trull and Ebner-Priemer, 2020). In addition to the measurement heterogeneity induced by the use of disparate items, this also creates additional barriers to replication.

### Replicability

There have been several efforts to create standardized reporting guidelines for EMA studies (Kirtley et al., 2019; Vachon et al., 2019). One such project is the ESM Item Repository project (https://osf.io/kg376/), which was created, in part, due to the fact that a large number of published EMA studies do not report the exact items used to study their constructs of interest. In the long run, failing to transparently report these methodological details could be detrimental to the replicability of this burgeoning field of research. As noted by Stone and Shiffman (2002), a lack of transparency around these methodological specificities for EMA studies was already notable in the early 2000s. While it was state of the art in other research fields to report relevant information, many EMA studies did not follow suit (Stone and Shiffman, 2002).

Almost 20 years later, this problem is still highly relevant. Since then, other researchers such as Kirtley et al. (2019), have begun studying these shortcomings. As recently as 2019, this group of researchers found that open science practices were only rarely implemented in EMA research (Kirtley et al., 2019). Furthermore, Trull and Ebner-Priemer (2020) reported in their systematic review of EMA studies, that less than a third of papers reported the psychometric properties of the items used, as well as the origins of the selected items. Similarly, approximately a third of the papers included in this systematic review provided a full list of items and the corresponding response scales and temporal contextualizations (Trull and Ebner-Priemer, 2020). The fact that transparent reporting in EMA designs appears to be a widespread problem, this must be rectified. Since replicability relies on providing such information in a clear and transparent manner, studies that fail to disclose this information limit the ability of other researchers to provide further support for their findings.

### The Current Study

The present study provides a review of measurement designs in EMA studies of mood and anxiety symptomatology among adults. Mood and anxiety symptoms were selected due to the high prevalence of disorders such as MDD and GAD in the general population and their significant comorbidity with each other (James et al., 2018; Kim, 2020). Mood and anxiety symptoms also form the primary focus of most EMA studies (aan het Rot et al., 2012; Walz et al., 2014). The goal of the current study is to provide an overview of how past EMA research has studied mood and anxiety symptomatology, point out the extent to which this information has been inconsistently reported, and provide guidance for future research in the form of a paper-by-paper item-specific database of how EMA studies have been designed in this area in the past. More specifically, we provide an outline of how frequently particular items were used across studies, how they were temporally contextualized, what response scales were used, what data collection platforms were used, how long participants were assessed for, and how frequently participants were contacted per day. We also provide item-specific reporting guidelines in order to encourage transparency in EMA research for mood and anxiety symptomatology moving forward.

## Methods

### Eligibility Criteria

This review sought out studies that fulfilled the following inclusion criteria: (1) the study had to investigate mood and/or anxiety symptoms, though studies asking about sub-threshold symptomatology were also be considered; (2) adult participants (over age 18); (3) EMA methods (also referred to as experience sampling, ESM, or daily diary methods) had to have been used; (4) the paper had to be in English. Studies were excluded from this review if (1) the sample included people experiencing psychotic or delusional symptoms, as well as (2) those dealing with substance use disorders. Papers that only included passive data collection via actigraph without subjective ratings of mood or other symptoms were excluded as well. In order to provide a more exhaustive overview of the use of these methods, no lower time constraint was placed and studies published up until April 2019 were included. Gray literature (i.e., dissertations) and unpublished studies (i.e., preregistrations, preprints) were included as long as they met the eligibility criteria described above.

### Information Sources and Search Strategy

MEDLINE, PubMED, and APA PsycNET databases were searched for published studies and poster presentations. OSF was searched for relevant preprints. OpenGrey was used to search for other gray literature. If identified studies met the eligibility criteria above, but information related to the specific EMA items administered was missing, the corresponding author was contacted. If no response was received within 1 month of contact, this information was marked as missing in the review. As mentioned previously, all studies published by the end of April 2019 were included in this review. Relevant research cited in the final selected studies (which may have not been identified through the initial search) was also screened for inclusion.

The following search terms were used for all databases (with only minor alterations to accommodate for differences across databases): (“diary” OR “momentary assessment” OR “experience sampling” OR “event sampling” OR “EMA” OR “ESM”) AND (“anxiety disorder^*^” OR “phobi^*^” OR “panic disorder” OR “PTSD” OR “post-traumatic stress disorder” OR “obsessive-compulsive disorder” OR “acute stress disorder” OR “agoraphobi^*^” OR “OCD” OR “GAD” OR “affective disorder^*^” OR “mood disorder^*^” OR “bipolar disorder^*^” OR “major depression” OR “MDD” OR “dysthymia”).

### Study Records

All identified studies were imported directly to Citavi. All initially-identified studies were screened for duplicates, and then for inclusion. Two members of the research team performed the two-step screening for relevance, first based on the papers' titles and abstracts, then based on the full text. For studies which the two authors drew different conclusions about inclusion on, discussions were held until a consensus was reached.

Next the full-texts of the remaining studies were obtained and uploaded to Citavi. They were then independently assessed by two reviewers (the first author of this protocol and another trained research team member) based on the eligibility criteria above. Each reviewer recorded why a particular study was excluded. After all identified studies were assessed by both reviewers, inter-rater agreement was calculated. For studies that the reviewers disagreed on, the article was discussed until consensus was reached (reviewing inclusion/exclusion criteria, and documenting disagreements and points of concession where relevant, mark as a borderline if relevant). The final number of articles excluded at this stage, broken down by reason for exclusion, was recorded. Full instructions provided to the team are available at https://osf.io/m8jsf/ under “Rater Guides.”

**Figure 1:**
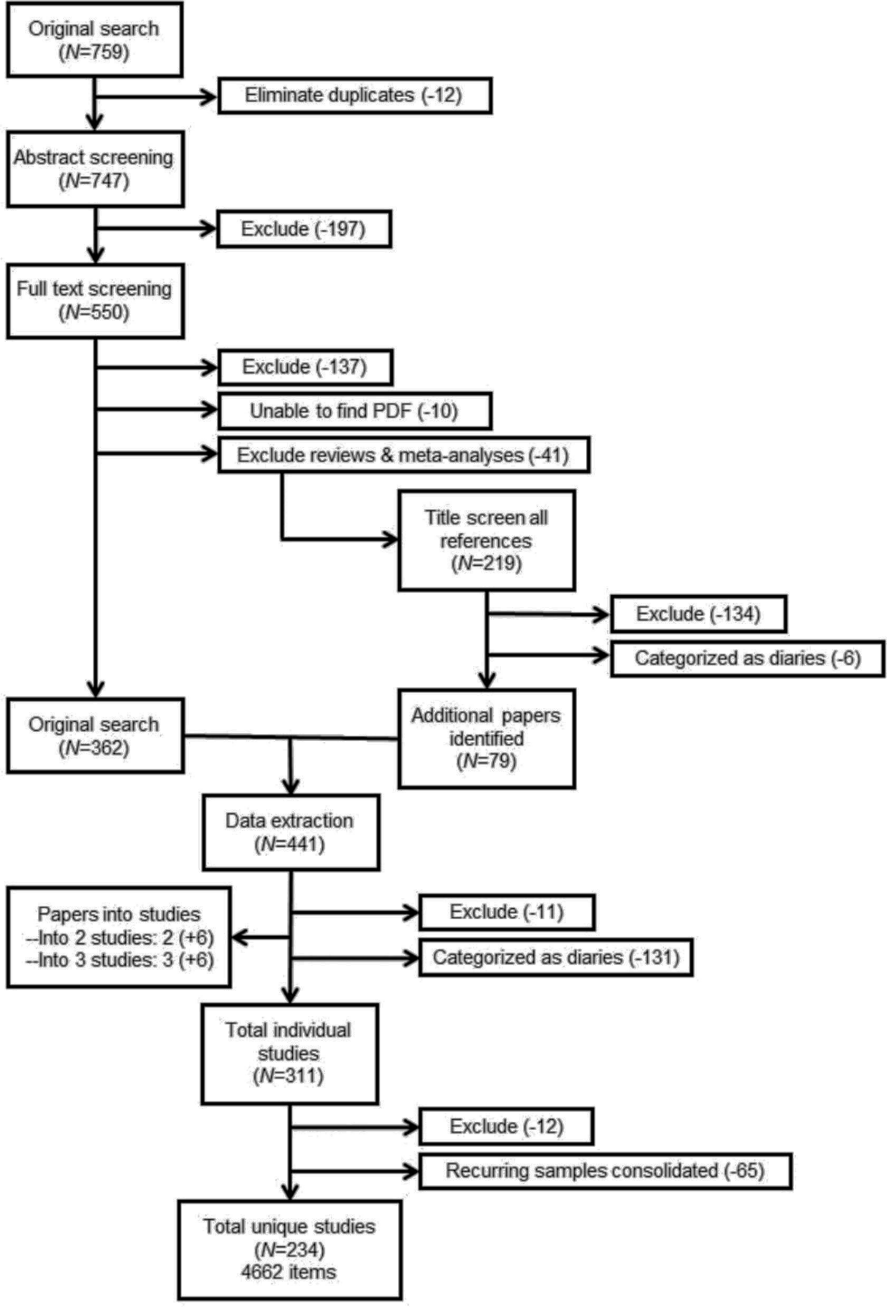
PRISMA flowchart. Total item count includes items that were unspecified or missing in-text. Further explanation of the full sample size can be found in the section Results.

The final sample of studies included then underwent data extraction. Each paper was read and relevant information (described in more detail below) was documented for each article. For clarity purposes, data was extracted directly into a Microsoft Excel file. The finalized document containing all extracted data is available at https://osf.io/m8jsf/ in the “Full Data Extraction” Excel file. Authors of included papers who wish to submit alterations to the data extraction file may contact the corresponding author of this paper. Updated versions with any requested alterations will be dated and published alongside the original data extraction file. For full details of the papers included and excluded throughout this process, see Figure 1.

After the initial data extraction, all information was checked by another team member, with a focus specifically on missing items and on studies that did not report their items but instead referred to standardized questionnaires (e.g., PANAS). Once again, the full instructions provided to the research team can be found at https://osf.io/m8jsf/ in the “Rater Guides” folder.

### Data Items

The following relevant information was extracted from each included study: (1) Work's citation; (2) Whether a mood/anxiety symptomatology studied was diagnosable, subthreshold, or a mix of both; (3) Type of EMA data collection platform (e.g., app, email, text); (4) For diagnosable mood/anxiety symptomatology, if there was any treatment administered and when in relation to the treatment the EMA data was collected; (5) Total duration of EMA data collection (in days); (6) Number of pings per day and how ping times were determined; (8) Construct/symptom measured; (9) Name(s) of scales administered (if using an established scale); (10) Full list of temporal contextualization for items (e.g., “since the last ping,” “in the last x hours,” etc.); (11) Full list of items administered; (12) Full list of response scales used (e.g., sliding scale, Likert, etc.); (13) Reliability indices (specify type, if included); (14) Validity measures (specify type, if included).

### Synthesis

In order to perform a qualitative analysis of the extracted data three members of the research team were trained in constant comparison and classical content analysis for this procedure (Leech and Onwuegbuzie, 2007, 2008). This team of three was composed of a doctoral student, and two departmental research assistants. Their training was conducted by the doctoral student and included a detailed description and step-by-step instruction on how to perform a qualitative analysis. This training included an overview of the widely used approach in qualitative research of first coding independently and then discussing and debating the resulting codes until a group consensus is reached. Consensus forms the basis for multiple qualitative methods, such as Consensual Qualitative Research (Hill et al., 2005), and ensures that various worldviews and perspectives have been taken into account when conducting this kind of research. Items from all studies included in the systematic review were analyzed qualitatively, using a Qualitative Description framework. The goal of this approach was to formulate a rich, succinct description of categories of EMA questions (Neergaard et al., 2009). All items were coded into categories by three independent coders and then discussed and consolidated to form a consensually agreed-upon list of categorized items. Due to the volume of items extracted, these consensus meetings took multiple weeks. Once all items were assigned to an agreed-upon category, each team member independently assigned categories to fit under broader themes, referring back to the original items when necessary. Once again a consensus group meeting took place in order to discuss and finalize the themes, their definitions, and the categories/items assigned to them.

The purpose of this analysis was to be able to systematically organize items across studies in order to more succinctly summarize findings. Numerical frequencies were assigned to capture how frequently items assigned to a specified category and theme emerged, in the style of classical content analysis (Leech and Onwuegbuzie, 2007).

## Results

### Systematic Review

Of the papers screened, 234 met our inclusion criteria and data were extracted as described above. Interrater agreement during the inclusion/exclusion process was high (*k* = 0.81), and all disagreements were resolved through discussion and review of the paper in question until a consensus was reached. The majority of these papers were published between the years 2011 and 2019 (*N* = 160, 68.38%) whereas only five papers (2.14%) fall into the period of the early 1980–1990. The remaining 69 papers (29.49%) were published between 1991 and 2010.

#### Population Studied

Regarding the populations studied, we found that an equal number of studies had samples with subthreshold (i.e., healthy samples; *N* = 88; 37.6%) and diagnosable symptomatology (i.e., patients with diagnosed mood/anxiety disorders; *N* = 88, 37.6%). For the purposes of this study, studies were categorized as having diagnosable cases if these were assessed using clinical interviews and/or self-report measures with clinical cut-offs. The remaining 58 papers recruited samples that contained either mixed cases (i.e., a group with diagnosable MDD and a healthy control group; *N* = 42) or random samples (i.e., where MDD was not screened for, but some diagnosable cases were more likely than not included; *N* = 16).

Overall, 130 papers included samples with diagnosable mood and/or anxiety disorders (55.56%). Of those 130 papers, 81 of them studied mood disorders (62.31%), 36 studied anxiety disorders (27.69%), while the remaining 13 studied a combination of both (10%). The most common diagnosis among the clinical samples studied was MDD (*N* = 48, 36.92), while only eight of the aforementioned 130 papers with diagnosable samples focused on GAD (6.15%). For further details on the specific diagnoses within the 130 papers with diagnosable conditions, see Table 1.

**Table 1:** Populations studied (Diagnosable only).

**Subthreshold**	**Mixed**				**Diagnosable**
**88**	**58**				**88**
	**Mood**		**Anxiety**		**Both**
	**81**		**36**		**13**
	MDD	48	GAD	8	
	BD	16	PD	4	
	PMDD	2	PTSD	12	
	Multiple	14	SAD	6	
	Unclear	1	Multiple	6	

*Subthreshold refers to populations who would not meet diagnostic criteria for a mood and/or anxiety disorder (e.g., healthy controls). Mixed samples refer to papers wherein a diagnosable and healthy control group were used, or a random sample was collected without conducting any diagnostics (thereby making it likely that, by chance, diagnosable cases were included in the sample). Diagnosable refers to samples that underwent some sort of diagnostic interview or tool and were determined as having significant symptomatology. The “Unclear” category refers to one paper, wherein specific diagnoses were not described, but participants had recently attempted suicide. “Multiple” refers to papers that included populations with several diagnoses within either the mood or anxiety disorders. MDD, Major Depressive Disorder; BD, Bipolar Disorder I/II; PMDD, Premenstrual Dysphoric Disorder; GAD, Generalized Anxiety Disorder; PD, Panic Disorder; PTSD, Post-Traumatic Stress Disorder; SAD, Social Anxiety Disorder*.

#### Treatment

Of the 234 papers included in this review, 46 (19.66%) included some form of treatment as part of the study. Furthermore, approximately half of those 46 (*N* = 21; 45.65%) conducted the EMA data collection exclusively at the same time as some sort of psychotherapeutic treatment. In just under a quarter of these 46 papers, EMA data collection was conducted both before and after treatment (*N* = 10; 21.74%). The remaining papers conducted the EMA data collection before (*N* = 2; 4.35%), after (*N* = 1; 2.17%), before and during (*N* = 6; 13.04%), during and after (*N* = 1; 2.17%), or before, during, and after (*N* = 5; 10.87%) some sort of psychotherapeutic treatment.

#### Data Collection Platform/Type

The data collection was carried out with the help of a variety of different devices and methods. The most common data collection method was the use of a portable device, such as a PDA or beeper (*N* = 67; 28.63%), followed by a paper-pencil designs (*N* = 55; 23.50%) and the use of apps on smartphones (*N* = 37; 15.81%). In general, network- and internet-based data collection (i.e., portable devices, smartphone apps, online platforms, emails) were the most popular methods (*N* = 134; 57.26%). The use of combined methods (i.e., by sending PDA reminders to fill out paper-pencil questionnaires), appeared regularly as well (*N* = 25; 10.68%). The rarer forms of data collection platforms were text messaging (*N* = 4; 1.71%), phone calls (*N* = 5; 2.14%), and email (*N* = 2; 0.85%). Of the 234 papers, seven reported using different types of data collection platforms throughout their study (i.e., having part of their sample use an app while the other uses paper-pencil; 2.99%), and four other papers failed to report this information at all (1.71%).

#### Response Scales

The response scales varied quite drastically across papers and even within studies. Of the 4,662 items extracted, the vast majority of them were measured using Likert scales (*N* = 3429; 73.55%). Among the Likert scales described, the amount of points available to be rated varied anywhere from 2 to 11 points. Within the pool of items rated via Likert scale, 5-point versions were most common (*N* = 1436; 41.88%), followed closely by 7-point variations (*N* = 1083; 31.58%).

Sliding response scales were also used for some items (*N* = 303; 6.50%). Though the heterogeneity in the number of points available in these scales was less pronounced than among the Likert scales, there were still some variations. Among the papers using sliding scales, they most commonly ranged from 0 to 100 (*N* = 130, 42.90%), though ranges of 1–100 (*N* = 50; 16.50%), 0–10 (*N* = 8; 2.64%), 1–5 (*N* = 4; 1.32%), and 1–7 (*N* = 30; 9.90%) were also used.

Multiple choice/checklist and open text entry style questions were equally uncommon, each being put to use 75 times among this pool of items (1.61% each). Simple Yes/No questions were administered for about 4% of the items (*N* = 197). Approximately 12% of the items were not accompanied by clear explanations about what kinds of response scales were used (*N* = 580).

#### EMA Duration and Pings Per Day

**Figure 2:**
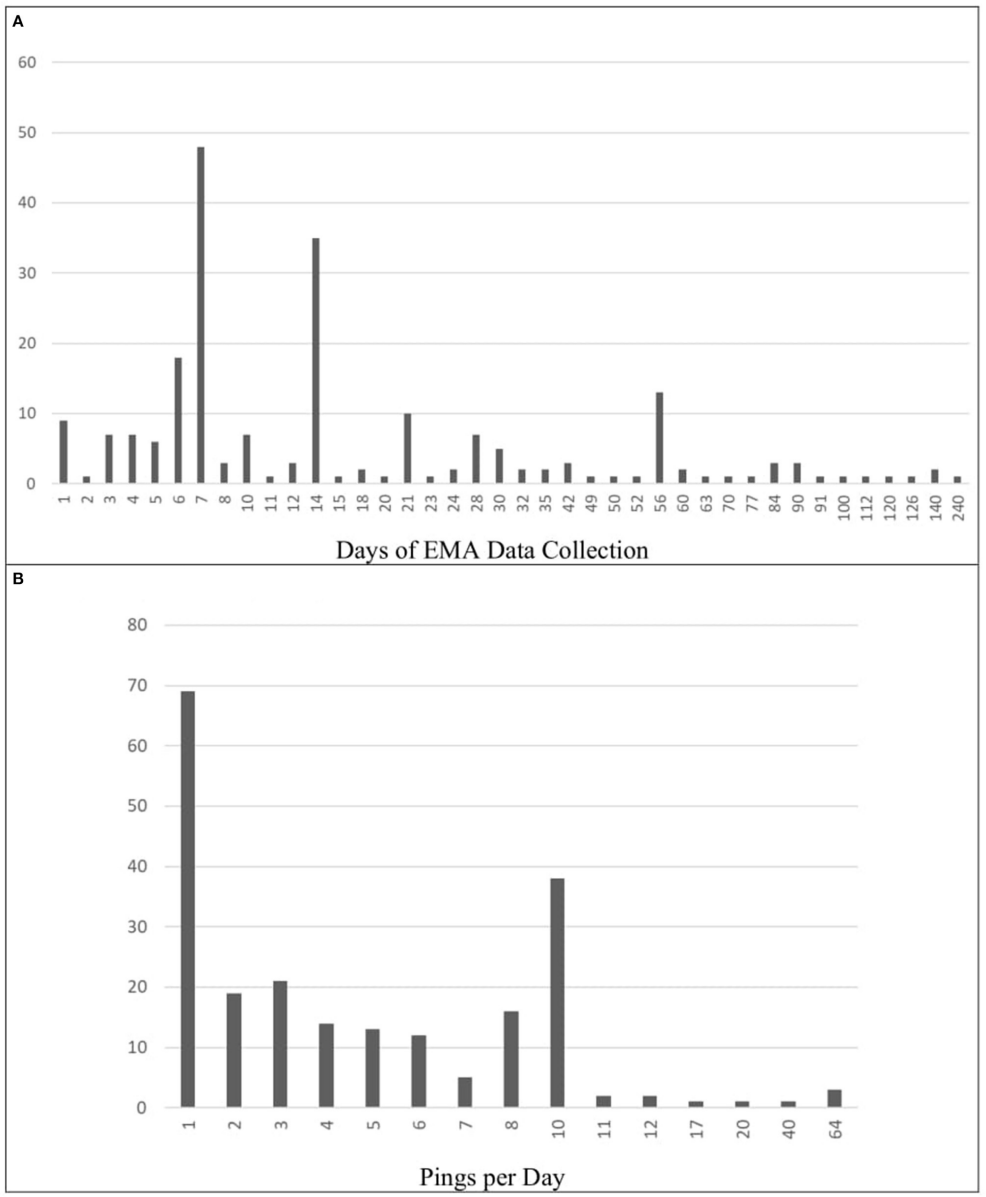
Frequencies of data collection schedules (Total days and Pings per day). **(A)** Frequency of Total EMA Data Collection (in Days). **(B)** Frequency of Pings per Day. Panel A shows the how many days of EMA data collection were used throughout the selected papers. In some papers, this information was either missing or unclear (*N* = 18). Panel B displays how many times per day participants were pinged across these same papers. All papers included in the review reported pings per day.

The duration of these EMA studies varied across papers, ranging from 1 to 240 days of data collection. Most of the studies determined the duration based on a particular number of weeks, meaning that a pattern of 7-day increments was quite typical. Most studies therefore lasted for 7 days (*N* = 48; 20.51%) or 14 days (*N* = 35; 14.96%). A small portion of studies also lasted 6 days total (*N* = 18; 7.69%) Across studies, the mean duration of EMA data collection was approximately 22 days (*M* = 22.79; *SD* = 30.53). This information was missing or unclear in 18 papers (7.69%). These results are depicted graphically in Figure 2A.

The mean number of pings per days was ~5, though this varied greatly across papers (*M* = 5.63; *SD* = 8.23). A large proportion of the papers pinged their participants once per day (*N* = 69; 29.49%) or 10 times per day (*N* = 38; 16.24%). Other ping frequencies were used less often: two per day (*N* = 19; 8.12%), three per day, (*N* = 21; 8.97%) four per day (*N* = 14; 5.98%), five per day (*N* = 13; 5.56%), six per day, (*N* = 12; 5.13%), seven per day (*N* = 5; 2.14%), and eight per day (*N* = 16; 6.84%). Frequencies above 10 were applied in only 10 papers (4.27%). In exceptional cases, participants were pinged 64 times per day (*N* = 3; 1.28%). These results are depicted graphically in Figure 2B.

Event-contingent reporting was used 15 papers (6.41%). Only four of these papers relied exclusively on event-based responses (1.71%).

#### Reliability and Validity

Psychometric properties were very rarely reported, with 134 papers (57.26%) reporting neither reliability nor validity indices for the EMA questionnaires used. Reliability was reported alone in 72 papers (30.77%) while validity was reported alone in seven (2.99%). Both validity and reliability indices were reported in 21 of 234 papers (8.97%) included in the present systematic review.

#### Temporal Contextualizations

A variety of different temporal contextualizations were used across the 4,662 items extracted. Momentary contextualizations (e.g., “right now,” “at the moment”) were used for 1200 of the items (25.74%). Contextualizations referring to a whole day (e.g., “over the course of the day,” “today”) were used for 632 items (13.56%). Some items referred participants to think about the moment right before the signal (e.g., “before the beep,” “shortly before the prompt,” *N* = 75; 1.61%). Event-specific signals (e.g., “after the stressful event,” “during your last interaction”) were also reported (*N* = 181; 3.88%). Intervals referring to the time since the last EMA report appeared 151 times (3.24%). Some prompts referred to more specific recent time-frames including the last half-hour (*N* = 8; 0.17%), the last hour (*N* = 53; 1.14%), the last 2 h (*N* = 4; 0.09%), the last 3 h (*N* = 23; 0.49%), and the last 4 h (*N* = 2; 0.04%). More often than not, exact descriptions of the temporal contextualizations for the EMA items were described unclearly or entirely missing (*N* = 2,103; 45.11%).

### Qualitative Analysis

In the qualitative analysis after reaching consensus 274 categories were identified. Categories that were assigned to the highest number of items were happiness (174 items, 3.73%), tension (129 items, 2.77%), sadness (114 items, 2.45%), anxiety (97 items, 2.08%), and stressful event (94 items, 2.02%). Percentages here are based on the overall number of items, before removing those that were phrased ambiguously or only mentioned in passing (*N* = 4, 662 items).

The 274 categories were grouped into 12 themes: (1.) Behavior—questions related to action/reaction to a specific situation (e.g., self-harm, risky behavior) (2.) Context—questions related to the participant's surroundings (e.g., physical, social) (3.) Diary—repeated questions about a specific series of events that related more to the frequencies of that event occurring than on other aspects of that experience (e.g., sleep diary: when did you go to bed, when did you wake up, how long did you sleep, how many times did you wake up at night) (4.) EMA method—questions related to the EMA methodology (e.g., how much participant were disturbed by the beep) (5.) Emotion regulation—questions related to the response to particular emotional experiences (both adaptive and maladaptive) (6.) Event—questions related to anything the participant had experienced recently/currently (e.g., activities, most stressful event) (7.) Mood—related to psychological affective state (8.) Self-awareness—questions about the participant's appraisal of themselves (both positive and negative) (9.) Somatic—physical symptoms and experiences (e.g., bodily pain) (10.) Symptoms—other more specific symptoms that did not fall into the categories above (e.g., phobic reactions, unspecified symptoms of MDD) (11.) Thought—cognitive questions, like an internal monolog and (12.) Missing—fully missing items.

After excluding the items that were unclearly described or entirely missing (*N* = 983; 21.09%), or which utilized dichotomous/opposite terms (making coding more difficult; *N* = 54; 1.16%), the total number of items was reduced from 4,662 to 3,625.

The most prevalent among the identified themes focused on mood. 2,208 items fell under this theme, representing 60.91% of all qualitatively analyzed items. The mood theme contained categories such as *happiness* (e.g., “To what extent you have felt this: happy”), *annoyance* (e.g., “How strongly you have felt annoyed during the past 2 h?”), and *difficulties concentrating* (e.g.., “I had trouble keeping my mind on what I was doing”).

The event theme contained a smaller percentage of the analyzed items (*N* = 341; 9.41%), and included categories such as *positive event* (e.g., “Did something positive happen since the last assessment?”) and *stressful event* (e.g., “During the last day I felt burdened by work?”). More rarely, the context theme (*N* = 303; 8.36%), which included categories such as *social interaction* (e.g., “We are doing something together”) and *social isolation* (e.g., “I prefer to be alone”), and thought theme (*N* = 172; 4.74%), including categories of *rumination* (e.g., “at the moment I am thinking about my problems”) and *flashbacks* (e.g., “how often did you have negative memories or thoughts about the trauma today”) occurred. The self-awareness theme (*N* = 169; 4.66%) included categories such as *self-efficacy* and *mindfulness*, which were represented by items such as “I am successful in my current activity” and “I am focused on the present moment,” respectively.

**Figure 3:**
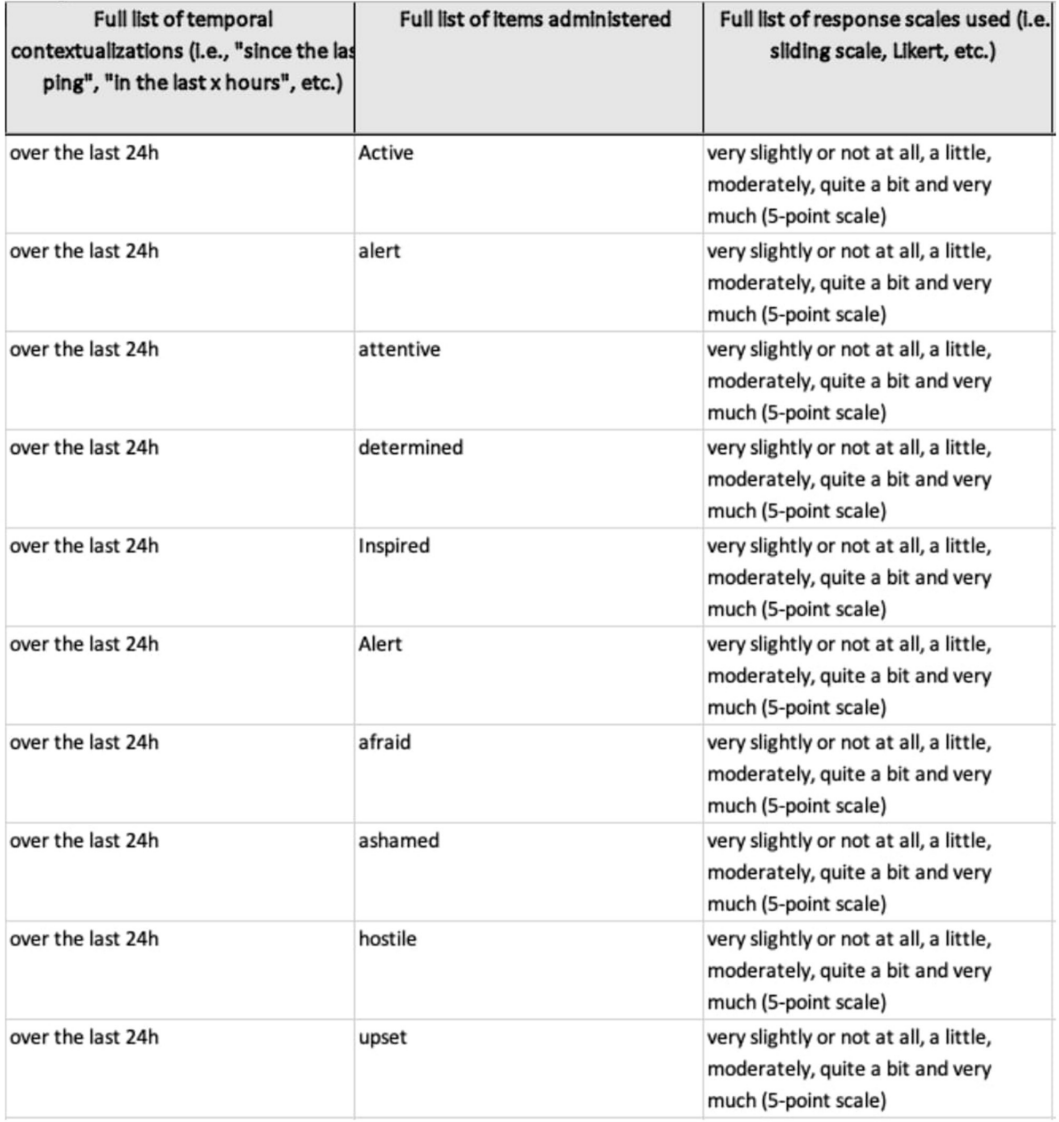
Insight into full data extraction database. Screenshot of part of the database, available at https://osf.io/m8jsf/. The full file includes all the information described within the section Methods. Inconsistencies in formatting or phrasing are the result of differing amounts of detail from paper-to-paper, different people having conducted the data extraction, or as a code for how the data was attained (yellow boxes indicate that the information was missing in the original paper, but were found in a different source, italics and square brackets indicate vague phrasing). Full details, including the instructions provided during data extraction, can be found in the Rater Guides on the aforementioned OSF page. The corresponding author will gladly update or add information from authors cited, if requested.

The remaining categories contained 431 items in total (11.89%). A full overview and paper-by-paper breakdown of these results, including the full lists of items and each of their qualitatively-identified categories and themes can be found at https://osf.io/m8jsf/ in “Full Data Extraction” file (for a preview of how the database looks like, see Figure 3). A list of all possible combinations of categories and themes, along with the associated frequencies is also available in the “All Possible Cat-Theme Combos” Excel file. Lastly, a full list of all items, organized by theme, can be found in the “All Items by Theme” Excel file.

## Discussion

This review systematically analyzed 234 EMA studies focusing on mood and anxiety symptoms. Overall, just over 62% of these studies included diagnosable cases on mood and anxiety disorders, with the remaining papers focusing on similar symptomatology among non-diagnosable cases. The vast majority of these diagnosable cases were mood disorders (~62%). A small portion of the included studies, just under 20%, included some form of psychotherapeutic intervention over the course of the study, and EMA data collection was most commonly conducted exclusively at the same time as the aforementioned intervention. The tools with which EMA data was collected varied as well, with portable devices (e.g., PDAs), paper-pencil designs, and smartphone apps being the most common in descending order. Likert-type scales were implemented most commonly across the items extracted, with 5-point scales emerging as the most popular variation. The total duration of EMA data collection varied significantly as well, though 7-day schedules and 1-a-day pings appeared most commonly. The vast majority (~57%) of the included studies did not report reliability or validity for their measures. Across the extracted items, momentary temporal contextualizations (e.g., “right now”) were used most commonly, in about a quarter of cases.

In order to describe and categorize the 4,662 extracted items, they were qualitatively analyzed. A system of 274 categories which fell under 12 broader themes was developed. These themes distinguished between questions related to specific behaviors, contextual factors, when they were presented in the form of diaries, questions that focused on the EMA method itself, how participants regulated their emotions, participated in specific events, their mood, sense of self-awareness, somatic experiences, their thoughts, and other more specific symptoms. The last theme was reserved for items that were insufficiently described or missing. Our findings suggest that even between studies that allegedly measured the same constructs, heterogeneity and differences in reporting of items leaves room for improvement (Stone and Shiffman, 2002; Vachon et al., 2019; Trull and Ebner-Priemer, 2020). This heterogeneity was to be expected, given that these studies each investigated these constructs from different angles and posed unique research questions. However, the degree to which many of the items were not disclosed or described only vaguely represents a large gap in the field. Transparency and clarity about these aspects of EMA papers should be encouraged in future research.

Extracted information can be found paper-by-paper at https://osf.io/m8jsf/, as well as a list of unique items used within each qualitative theme. We encourage researchers to use these databases as a resource to identify potentially relevant items for future studies. Additionally, we provide a template for reporting relevant EMA-specific measurement details, to be used and published in future research, or made available through online supplements. We hope that providing this framework for item-level reporting will help increase transparency and discourse around EMA methodology, and ensure that it continues to flourish in a scientifically replicable and sustainable way.

EMA remains a very flexible and highly adaptable research design. Though this flexibility allows for ample creativity and adaptability to different research questions (Janssens et al., 2018), the resulting heterogeneity means that drawing conclusions across studies is not always advisable. Although similar constructs, such as positive/negative affect or stress, were consistently measured throughout the identified studies, finding research that implemented identical measurement practices was incredibly rare. The most glaring gap in the EMA field is psychometric validity and reliability of items. In the present study, if studies reported previously calculated psychometric values (e.g., from the original validation studies), these were counted as having reported these values. However, this means that studies were coded as having reported psychometric values, although these were not always verifiably calculated based on the papers' own data. Many studies adapted cross-sectional questionnaires to better suit the EMA format, but then failed to provide either between- or within-person reliability or validity information for their adapted measures. Though these details may seem inconsequential, it must be noted that small differences in the methodology can have a profound impact on what participants report (Flake and Fried, 2019). Future research should distinguish between whether the psychometric properties reported were calculated based on the sample collected, or if they simple rely on previously published results. This distinction is essential when validity and reliability calculations do not take the EMA format into consideration.

In addition to the inconsistency with which item-specific information was disclosed, there were also variations in the type of device from which EMA data was collected. Although there is meta-analytic evidence for high correlations between paper- and computer-administered questionnaires (Gwaltney et al., 2008), the specific varieties of modern computer-administered questionnaires have not been explored in much detail. User interfaces vary between different apps, meaning that the user experience could be completely different from study to study. Also, the difference between receiving an email or text message reminder to fill out a paper-pencil questionnaire vs. using an app with push notifications may also have an influence on how participants report their symptoms. Given the large amount of EMA-specific apps on the market, in addition to numerous other methods such as paper-pencil data collection or phone calls, it is vital that the impact of the type of data collection platform used be studied in more detail.

In addition to the platform upon which EMA data is collected, response scales differed greatly across studies as well. Given that factors, such as the length of the Likert scales, can impact participants' responses (e.g., Dawes, 2008), it is imperative that EMA researchers select their response scales carefully. Likert scales emerged as the most common response scale, though the number of points on the scale varied. This variation occurred not only across papers, but within papers as well. Some studies used 5-point Likert scales for some questions, and 7-point scales for others. Proceeding to use this ordinal data, reaped from varied ordinal scales, to create sum scores and later analyze it as though it were continuous, represents another grave methodological and psychometric misstep. Sliding scales present a better choice for such continuous analyses. However, even sliding scales had slight variations: 0–100, 1–100, 1–101. Least problematic among these variations were the differences in multiple choice or text responses. These were used more pragmatically, when categorical responses were required (e.g., *Who are you with right now? Check all that apply: Family, friends, colleagues, alone*) or when it would have been impractical to provide a long list of options for participants to choose from.

Items were temporally contextualized with language such as “today,” “since the last beep,” “in the last 15 min,” and “right now,” all of which clearly aim to measure constructs at different moments in time: in the moment, within a specific time frame, or over the course of a full day. Though these differences may seem minute, participants may respond more accurately to some time-specific prompts than others (Stone et al., 2020). For example, participants seem to struggle to accurately report responses when “during the last 24 h” is used, whereas “today” or “since waking up today” seemed to improve the accuracy with which participants responded (Stone et al., 2020). Given these reporting differences, it seems likely that similar, if not more pronounced differences would exist across prompts referring to different timeframes. Overall, we recommend that EMA researchers examine the literature to justify what level of granularity they require to capture the constructs they are interested in. However, more research is required to determine the extent to which differences in prompts impact participant ratings, particularly for more granular time-frames (e.g., “since the last ping” vs. “in the last 2 h”). In the meantime, we hope that our database helps researchers find other groups who share similar research questions and may open a dialogue about best practices, depending on the research group's specific goals and interests.

Overall, a combination of signal- and interval-contingent reporting emerged as most common across the selected papers. The fact that fewer papers utilized event-contingent designs makes sense given the examined population of mood and anxiety symptoms. Both of these types of symptoms are, to a certain extent, governed by dynamical and fluctuating systems of symptoms and environmental factors (Cramer et al., 2016; Kossakowski et al., 2019). Thus, the ebbs and flows of the symptoms may be more difficult to capture with an event-contingent EMA schedule, unless they relate to something specific happening (e.g., a panic attack), though these types of specific events are uncommon with MDD and GAD (Ebner-Priemer et al., 2009).

The fact that the majority of the samples from selected papers had diagnosable mood disorders may explain why one of the main qualitatively-identified themes of items was mood-focused. However, meta-analytic findings show that studies using EMA with samples dealing with depression, PTSD, anxiety, and eating disorders tend to disproportionally include emotional items (Newson et al., 2020). More specifically, items related to fear, panic, and anxiety, as well as mood and outlook, appeared frequently in assessments of the aforementioned disorders, similarly to the findings from our qualitative analysis. The overlap between our results and the results presented by Newson et al. (2020) suggests that mood and affect are important factors across various disorders, and may not only have emerged as dominant themes due to the samples selected for this systematic review. There are innumerable ways in which such mood and affect constructs can be measured, once again, depending on the goals and interests of the researchers. Again, we hope that sharing our database will facilitate the decision-making process of identifying appropriate items, picking a suitable measurement schedule, and selecting a response scale.

One of the most striking findings of this systematic review were the inconsistencies in the information provided in selected papers: some papers provided full lists of items and response scales (or linked to supplemental materials were these were available), while others only provided vague descriptions of the construct measured (e.g., *we measured depression using three EMA items* without further elaboration). In their meta-analysis, Trull and Ebner-Priemer (2020) analyzed a total of 63 papers and collected the percentage of papers which followed recommended reporting criteria (e.g., “report full text of items, rating time frames, response options or scaling”). They found that 78% of the papers followed this recommendation. The present sample strongly supports this finding, because 917 of the total 4,662 items (19.67%) were unspecified, meaning that the phrasing of the item was missing in the underlying paper. At times, EMA items were not described at all or their phrasing was incomplete or vague, meaning that many items were not able to be included in the qualitative analysis at all. Whenever possible, for example if cross-sectional questionnaires were adapted for EMA use, the approximate wording was extracted from other papers or online resources. The database we provide contains as much information as we could reasonably assume or gather from published materials, once again, in an effort to promote transparency among EMA research and provide a resource to facilitate future EMA research design decisions.

Trull and Ebner-Priemer (2020) appeared to have similar difficulties with missing information: In their review only 17% of the studies provided information on the rationale for an EMA design or discussed their sampling density. In order to compare, replicate, and conduct meta-analyses it is crucial to report all relevant information, if necessary in the supplemental materials (due to journal restrictions). We therefore strongly advise future EMA researchers to provide item phrasing, psychometric properties and further materials relevant to the design (Appelbaum et al., 2018; Vachon et al., 2019; Trull and Ebner-Priemer, 2020). In addition to the item-specific database of past research, we also provide an item-level reporting template (https://osf.io/frxa7/) which, if used consistently moving forward, could greatly improve the transparency and replicability of EMA research for mood and anxiety symptoms.

### Limitations

This systematic review attempted to identify recurring samples (i.e., when a group of researchers used the same sample and therefore research design to publish multiple papers) in order to reduce any bias on the frequency with which certain research designs were implemented. The purpose of this was to avoid double-counting findings from larger and potentially more prolific labs. However, this task proved daunting, given the large number of papers and items extracted. One of the limitations of the present systematic review is therefore the fact that we cannot be sure whether we accurately and exhaustively excluded recurring samples. This points to a larger challenge in not only the EMA literature, but at the macro-level: identifying which papers belong to which larger projects is near to impossible without any personal knowledge. With the increasing push toward open science practices such as pre-registration, it may be useful to find a way to retro- and prospectively group papers belonging to the same umbrella data collection project (Asendorpf et al., 2013; Kirtley et al., 2019). Self-citation of previously published papers seems to have been a temporary and sporadically used technique for doing so. Moving forward, it may be helpful to group such papers using DOIs or using pre-registration IDs of some sort. Putting this type of system in place would additionally allow researchers to transparently share information about their data collection procedure that may edited out during the publication process.

Despite the fact that we did not set a lower limit for EMA papers to be included (identified studies were published between 1980 and April 2019), we acknowledge that several relevant papers have not been included in this review. Since April 2019, due to the widespread and ever-increasing use of technological devices (along with a recent increased interest in EMA-style data collection in general), the number of studies published after we performed our search has continued to increase (e.g., Hollands et al., 2020; Schoevers et al., 2020). Therefore, we acknowledge that our review is not exhaustive, and does not include a number of papers that have been published more recently. It is our hope that this paper's findings will be expanded upon to include these newer papers. We invite researchers to use our already extracted data, available at https://osf.io/m8jsf/, as a starting point for this important research.

We also acknowledge that the search terms used include a diagnosis, Obsessive-Compulsive Disorder (OCD), which is no longer considered a mood/anxiety disorder (American Psychiatric Association, 2013). However, papers which included OCD populations were not extracted as part of the diagnosable anxiety disorders, which we conceptualized according to the DSM-V (American Psychiatric Association, 2013).

The most considerable limitation of this review is the fact that we cannot make conclusions about compliance and retention in the context of EMA design decisions. It is our hope that this paper's findings will be expanded upon to include newer papers, as well as extracting additional information about the different EMA designs' compliance and missingness levels, as well as which kinds of analytic methods were used to analyze the EMA data. Our hope is that expanding the present review with this information will provide more practical guidance about which designs might boost compliance and suppress missingness most, and which statistical approaches can optimize power (i.e., Bastiaansen et al., 2020; Eisele et al., 2020). We invite researchers to use our already extracted data, available at https://osf.io/m8jsf/, as a starting point for this important research.

### Conclusion

Overall, our findings point to a striking gap in the EMA literature, namely the lack of transparent and clear documentation of how the design was chosen and implemented. There are many ways in which two seemingly identical EMA studies could, in actuality, differ. For example, they may state that they both study panic attacks using EMA: one might require participants to fill out brief 3-item, 0–100 sliding-scale questions about their current anxiety, current shortness of breath, and how likely they feel to have a panic attack; the other might measure the number of panic attacks someone has over the course of a week, asking once a day about whether a panic attack occurred and if so, under what circumstances. Clearly, these two studies differ quite significantly and comparing results from them would be questionable at best. However, unless the details about the ways in which EMA was implemented are reported, there is no way to determine whether or not such a comparison is reasonable. Therefore, transparency about design decisions, and careful selection of items and response scales will be essential to the continued use of EMA methodology.

Given the variation in designs used, we would like to impart on our readers that there is no exemplary design for EMA studies of mood and anxiety disorders. We urge researchers to use our database and the papers therein as inspiration for future studies. Additionally, readers may consult a study by Janssens et al. (2018) for an overview of the rationales for using different kinds of EMA designs, based on the judgment of various experts in this methodology.

Based on our findings, it is clear that there will not be a one-size-fits-all solution for improving EMA design. In fact, providing this kind of solution would be a disservice to advancing this area of research. However, we would like to make two tangible suggestions for EMA studies moving forward: (1) Clear, transparent reporting of research design, and (2) Thoughtful, theory-based selection and validation of EMA design components (including items, response scales, data collection schedules, etc.).

First, we encourage researchers to transparently disclose details about their EMA research design. This can be done in a number of different ways: publishing various details alongside the results in journals or (if space is limited) through pre-registration and/or use of an open access online supplement (see Kirtley et al., 2019). To facilitate this process, we provide a template for reporting such information on an item-specific level at https://osf.io/wa8u5/.

Second, we recommend selecting and wording EMA items thoughtfully, while also considering the impact of other EMA-specific design decisions (e.g., response scales, temporal contextualizations, etc.). This can be driven by theory, but can also be supported by gaining insight into the ways in which other researchers have studied their constructs of interest in the past. For this purpose, we offer our open-source database as a starting point for EMA research about mood and anxiety symptomatology. In addition to having a strong basis for the EMA design and items, we also encourage researchers to ensure the psychometric qualities of the items they choose by calculating reliability and validity measures for their selected EMA design.

Alongside the insight provided by our item-level database, these recommendations might help to ensure that EMA research in the field of mood and anxiety symptomatology can continue to flourish and progress.

## Data Availability Statement

The original contributions presented in the study are included in the supplementary materials available on the project's OSF page (https://osf.io/m8jsf/). Further inquiries can be directed to the corresponding author.

## Author Contributions

MH and JR were involved in the development the study concept. MH, PS, and YK performed the qualitative analysis. Rough drafts of this paper were written by PS and YK under the guidance of MH, though significant re-writing, reformatting, and preparation for submission was done by MH. JR provided critical revisions. All authors contributed to the study design, as well as the screening of papers, data extraction, and approved the final version of the paper for submission.

## Conflict of Interest

The authors declare that the research was conducted in the absence of any commercial or financial relationships that could be construed as a potential conflict of interest.
